# Comparison of the Chemical Composition of Six Canihua (*Chenopodium pallidicaule*) Cultivars Associated with Growth Habits and after Dehulling

**DOI:** 10.3390/foods12081734

**Published:** 2023-04-21

**Authors:** Jenny Mérida-López, Sander Jonathan Pérez, Rocío Morales, Jeanette Purhagen, Björn Bergenståhl, Cinthia Carola Rojas

**Affiliations:** 1 Food and Natural Products Center, San Simon University, Cochabamba, Bolivia; 2 Department of Food Technology Engineering and Nutrition, Lund University, Naturvetarvägen 12, 22362 Lund, Sweden

**Keywords:** Andean, ascending, canihua, decumbent, dehulling, growth habit, whole canihua

## Abstract

The canihua (*Chenopodium pallidicaule*) is a native Andean crop that stands out for its high content of protein, fiber, and minerals and that has a good fatty acid profile. We studied six canihuas cultivars, which were compared according to their proximate, mineral, and fatty acid composition. Based on the form of stems, termed growth habit, they belonged to two groups: decumbent (*Lasta Rosada*, *Illimani*, *Kullaca*, and *Cañawiri*) and ascending (*Saigua L24* and *Saigua L25*). Dehulling is an important process applied to this grain. However, there is no information about how it affects the chemical composition of the canihua. Dehulling resulted in two levels, whole and dehulled canihua. The highest protein and ash contents were in whole *Saigua L25* (19.6 and 5.12 g/100 g, respectively), and the highest fat content was found in dehulled *Saigua L25*, while the whole grains of *Saigua L24* presented the highest fiber content (12.5 g/100 g). Dehulling mainly affected the macro-minerals content, while micro-minerals were only slightly linked to the dehulling. The growth habit influenced the C18:1 and C18:3 contents. In conclusion, the canihua had a nutritional composition influenced by each variety, strongly influenced by dehulling, and to a lesser extent by growth habit.

## 1. Introduction

According to the World Health Organization (WHO), the COVID-19 pandemic exponentially increased the number of people affected by hunger and malnutrition, up to 768 million in 2020., In addition, the WHO suggests healthy diets protect against malnutrition in all its forms, including non-communicable diseases such as diabetes and cancer, ensuring adequate intake of macronutrients (proteins, fats, and carbohydrates, including dietary fiber) and essential micronutrients (minerals and vitamins) [1]. Based on this perspective, it is necessary to deepen the knowledge about certain crops with a suitable nutritional profile. 

Canihua, *Chenopodium pallidicaule*, is a nutritious grain from the South American Andean highlands [2], cultivated intensively around Lake Titicaca in La Paz, Bolivia, and Puno, Peru, under “altiplano” conditions, i.e., frost, drought, and saline soil [3], with an altitudinal distribution range between 3200 and 4200 masl [4]. 

**Figure 1:**
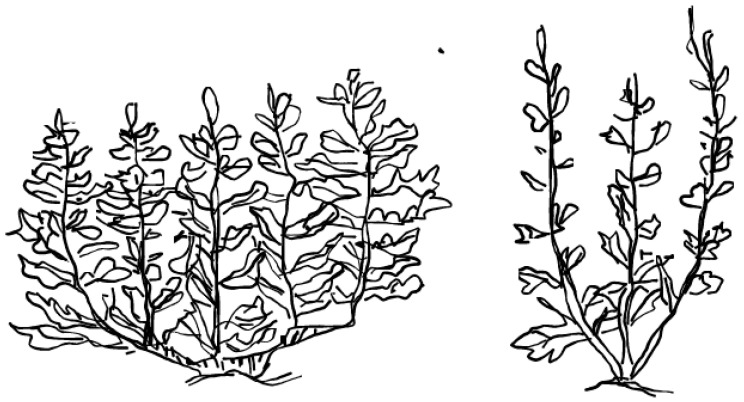
Growth habit, decumbent/*lasta* (**left**) and ascending/*saigua* (**right**). From Tapia et al. [6].

Canihua presents a wide number of varieties and cultivars [2,5]. The physical form of the stem in the plant is part of the growth architecture of the plant, termed the growth habit [6], and it could be used to differentiate the varieties from each other, like in rice [7]. Canihua, by its growth habit, is divided into two groups, decumbent/*lasta* and ascending/*saigua*. The schematic shapes of the plants are shown in Figure 1 [6], although these growth habits have been called by other names, for example, prostrate and erect, respectively [8,9]. In addition, the architecture of the plants could be related to the fixation of nutrients, e.g., phosphorous fixation [10]. 

Cultivar is termed a set of plants that (a) has been selected for a particular trait or combination of traits, (b) is distinct, uniform, and stable in those traits, and (c), when propagated by appropriate means, retains those traits, according to the international nomenclature for plant crops [11].

Canihua and quinoa belong to the same *Chenopodiaceae* family, but canihua has negligible saponin content and can be consumed unwashed like whole grain [3]. For decades it has been well known that canihua is rich in macro and micronutrients, i.e., it has a higher protein content than amaranth and quinoa [2] with a biological value due to its balanced amino acids comparable to milk [12]. This aspect is positive for its inclusion in foods, including for vegetarians and vegans [12,13]. Nevertheless, the protein content and proximal content could differ between cultivars, as found in a previous study [14].

The hull of canihua grains is removed by dehulling to decrease the amount of saponins for international trade [15]. In pseudocereals, dehulling could also reduce the content of some micro-nutrients or minerals such as phosphorus and calcium [16]. Calcium, iron, and zinc are minerals with a higher content in canihua than in other cereals such as corn or wheat [17]. Studies have reported its antioxidant capacity [18], fiber content [5], and fatty acid composition [19]. However, no study has presented the effect of dehulling on canihua grain, which is traditionally consumed like whole grain [14]. Therefore, analysis of dehulled and whole canihua grains is needed.

The commercial cultivars of canihua have different growth habits, but no previous study has evaluated this effect on the nutritional composition. Furthermore, as mentioned previously, this grain is eaten whole and dehulled without knowing the nutritional implications. We hypothesize that cultivars, growth habits, and dehulling may affect the nutritional composition, proximate, mineral, and fatty acid composition of canihua grains.

## 2. Material and Methods

### 2.1. Samples and Samples Preparation

Six canihua (*Chenopodium pallidicaule*) cultivars were obtained from Fundación para la “Promoción e Investigación de Productos Andinos”, PROINPA, a foundation for promoting and supporting research of Andean products. The material was harvested in 2019 and delivered in samples of 2 kg each. Four samples displayed a decumbent habitat (*Lasta Rosada*, *Illimani*, *Kullaca*, *Cañawiri*) and two an ascending habitat (*Saigua L24* and *Saigua L25*) (Table 1). The grains came from 3 different locations: Caquiaviri, Khipakhipani, and Patacamaya at 3900, 3857, and 3700 m above sea level (masl), respectively. The cultivars, origin, cultivation altitude, growth habit, growth cycle, and yield are summarized in Table 1. 

**Figure 2:**
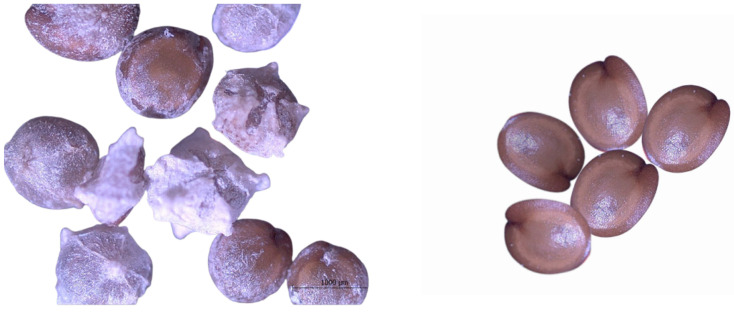
Canihua, whole grain (**left**), and dehulled grain (**right**), bar 1000 µm.

The grains were cleared from stems, leaves, and small stones before being separated into two samples. The first part was packed and stored at 4 °C, and the second was dehulled by manual friction and washed with running water (4–5 times) until pearl grains were obtained before being dried at 30 °C for 12 h, packaged, and stored at 4 °C. There were twelve samples, six whole grains, and six dehulled grains. Images of whole and dehulled grains under a microscope (Leica DM 2000, Wetzlar, Germany) are shown in Figure 2.

Each sample was milled (Retsch, ZM 200, Hann, Germany) at <0.5 mm and <1 mm for chemical and mineral analyses. The obtained flours were packaged and stored at 4 °C until further analysis.

### 2.2. Proximate and Composition

Proximate analysis was evaluated using the AOAC official methods [20]. Moisture content (AOAC 925.10) was determined by drying approximately 2 g at 105 °C until reaching a constant weight. The protein content (AOAC 984.13) was determined using the micro-Kjeldahl method with a protein factor of 6.25 to obtain the protein content from the total nitrogen. The total lipid content (fat) was determined by Soxhlet hexane extraction (AOAC 963.15). Ash content (AOAC 923.03) was analyzed by ignition at 550 °C until light gray ash was obtained. The crude fiber content was determined by acid-alkali hydrolysis (AOAC 962.09). The carbohydrate content was estimated by the difference.

$$C_{carbohydrate}=1-C_{protein}-C_{lipids}-C_{fiber}-C_{ash}$$

where $C_{component}$ refers to the mass fraction of the component count on the total dry matter.

The starch content was determined using an enzymatic total starch kit (Megazyme International Ltd. Co, Wicklow, Ireland), as described by Mérida et al. [21]. 

### 2.3. Mineral Content Determination

The content of macro (Ca, K, Mg, and Na) and micro minerals (Cu, Fe, Mn, and Zn) was obtained by wet digestion of approximately 0.5 g of sample with 5 mL of hydrochloric acid and 3 mL of hydrogen peroxide in a microwave followed by atomic absorption measurements. The P content was obtained by extracting the ash and spectrophotometric analyses using a molybdenum complex [21].

### 2.4. Color

The color of the grains was evaluated using the color “space”, usually referred to as the 1976 CIELAB color space (established at the 1976 CIE congress) with a colorimeter (KONICA MINOLTA, Chroma Meter CR-400, Tokyo, Japan. The CIELAB system uses three dimensionless colorimetric parameters L*, a*, and b*. Although in a simplified form, L* represents the brightness with 0 for black and 100 for white, a* represents the opposite colors green (negative values) and red (positive values), and b* represents the color range between yellow (positive values) and blue (negative values). This system is more challenging to understand under a space than a single point. Origin (the intersection of a* and b*) represents the white point [22]. 

### 2.5. Fatty acid Composition

The extraction, derivatization, and analysis by gas chromatography of fatty acids were conducted as described previously [21]. Oil was extracted by a chloroform/methanol (2/1, *v*/*v*) solution. The derivatization conversion was performed with methanol under alkaline conditions. The fatty acids were separated and quantified using a GC–FID system 7890B (Agilent Technologies, Wilmington, DE, USA) equipped with a Rt^®^-2560 capillary column (100 m × 0.25 mm × 0.20 µm; Restek, Part N° 13199, Bellefonte, PA, USA). The oven temperature began at 140 °C for ten minutes, and then increased at 3 °C/min to 240 °C. A standard of C4–C24 (Supelco, Bellefonte, PA, USA) unsaturated FAME mix was used.

### 2.6. Statistical Analyses

All analyses were performed at least in triplicate. Results are expressed as means with standard deviations (SD). The one-way ANOVA followed by Tukey’s with the SPSS Statistics 24 package (SPSS Inc., IBM Corporation Armok, IL, USA) was used to evaluate possible significant differences. Comparisons were made between the 12 samples, where the lowercase letters show the significant differences amongst cultivars in all grains, whole and dehulled, while the uppercase letters show significant differences amongst cultivars or growth habits in each group, whole or dehulled grains. The Unscrambler^®^ X 10.2 software (CAMO Software AS, Oslo, Norway) was used to perform principal component analysis (PCA). PCA is used as a statistical tool to visualize sample groups and identify significant underlying relationships.

## 3. Results

### 3.1. Proximate Composition

The proximate composition results are shown in Table 2. The cultivars differed randomly by protein and fat content. Dehulling did not change the protein content of the cultivars, while the fat content changed randomly. The ash content had a significant association with the growth habit in dehulled grains. The fiber content of *Saigua L24* is higher than that in the *Saigua L25* and the decumbent cultivars. The ash content decreased relatively with the dehulling, while the carbohydrate content increased. In dehulled grains, growth habits differed in starch content, with the lowest value for ascending cultivars.

**Figure 3:**
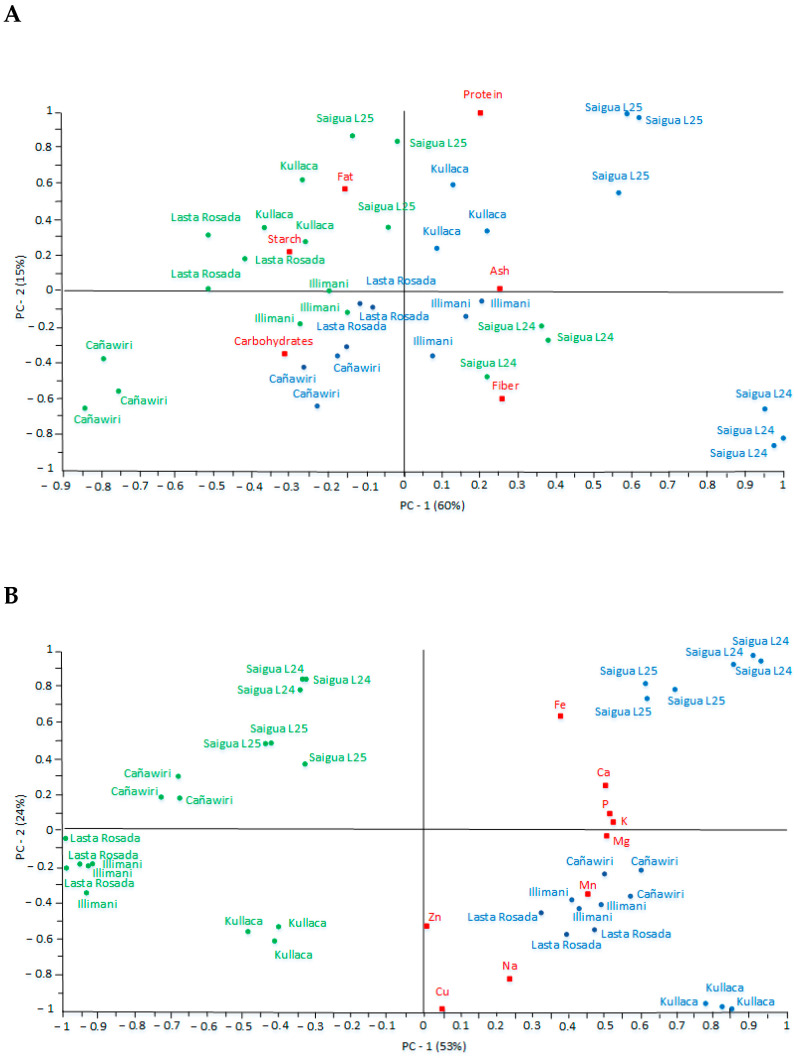

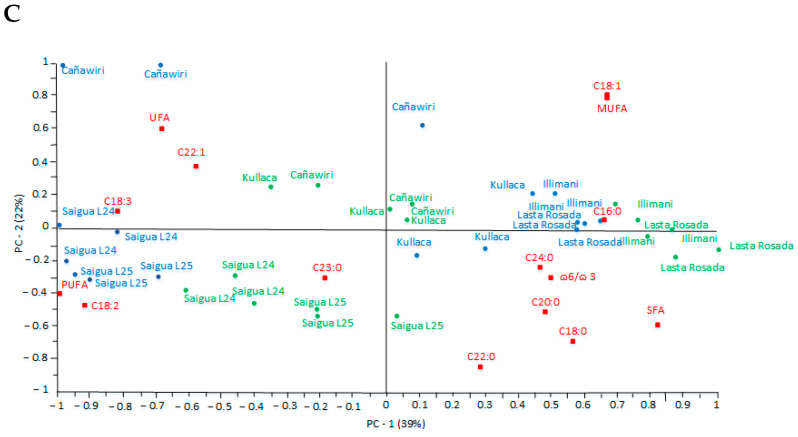
Principal Component Analysis (PCA) biplot of six cultivars of whole and dehulling canihua (*Chenopodium pallidicaule*). (**A**) Proximate composition. (**B**) Mineral composition. (**C**) Fatty acid composition. Blue: whole grains, green: dehulled grains.

Principal component analysis (PCA) in Figure 3A showed 75% of the explained variance, PC—2 divided the whole (right) from the dehulled (left) grains, with the exception of *Saigua L24*, where even the dehulled grain is within the whole-grain group. The composition of the dehulled grains differs distinctly from whole grains. 

### 3.2. Mineral Composition

The whole grain gave higher values for all the macro-mineral contents (P, K, Ca, and Mg), with minor variation for Na and the most significant variation for Ca and K (Table 3). In addition, the cultivar determined the P and K contents in the whole grain, and these differences were less pronounced in dehulled grains. The various cultivars have a similar Mg content in all whole grains. The whole ascending grains exhibited the highest Ca content, while their Na content was the lowest. Dehulling has a minor effect on microminerals (Zn and Cu, while most of the other minerals are strongly reduced by the dehulling process.) 

PCA analysis for minerals (Figure 3B) explained 77% of the explained variation of the dataset. It showed an effect due to dehulling, where two groups were distinguished by PC — 1, dehulled canihua (left) and whole canihua (right). In addition, PC — 2 divided the decumbent habits from ascending ones, except for dehulled *Cañawiri*. In Figure 3B, the Fe content is more correlated with the whole grains of the parent cultivars; these cultivars were also slightly correlated with the P, K, and Ca contents. The factors evaluated, dehulling, cultivar, and growth habit, did not affect the Cu or Zn contents. 

### 3.3. Color

The color parameters of all the grains are presented in Table 3. The whole grains are lighter and orange, while the dehulled flours are darker and yellowish. They are shown by the lower luminosity (L*) in the dehulled group. The values of a* increase after dehulling. The values of b* decrease after dehulling. The cultivars *Cañawiri* and *Saigua L25* have a stronger color than the other cultivars.

### 3.4. Fatty Acid Composition

The lipids are dominated by linoleic acid (18:2), with contents above 50% of the total FA for all the samples. The variation between the cultivars is slight, and the dehulling factor only leads to small changes in the composition. However, the variation between the cultivars (Table 4 and Figure 3C) seems to follow a pattern. Samples with lower levels of palmitic (16:0) and oleic (18:1) have higher linoleic (18:2) and linolenic (18:3) contents. The growth habit affected the FA profile for these cultivars, having the highest proportion of C18:3 and lowest of C18:1 for ascending cultivars. This effect was even extended for monounsaturated FA (MUFA) and polyunsaturated FA (PUFA). It was also observed that there are several comparably long saturated FA (arachidic (20:0), behenic (22:0), tricosanoic (23:0), and lignoceric acid (24:0)) present in small amounts.

## 4. Discussion

Canihua has a somewhat lower starch content than most cereals, which makes the protein content (up to 20% of Dry Mass (DM)) and lipids content (up to 10% DM) higher when compared to common cereals such as rice, corn, barley, and pseudocereals such as amaranth or quinoa [5,23,24]. Cultivar, dehulling, and to some extent maybe also the growth habit influence the protein content, which gave up to a 7% difference in protein content amongst the samples, where the whole *Saigua L25* (ascending) was found to have the highest content (19.6 g/100 g), while *Cañawiri* cultivar had the lowest content (13.9 g/100 g); this is normal variation according to other studies (14.1–18.8 g/100) [12,14,25]. The protein content of amaranth has been found to be directly related to the growth altitude [26]. 

In this study, the growth habit in terms of plant shape did not take into consideration the growing location or any genotype, environment, soil, or harvesting conditions. It is well known that these conditions can also have an impact on growth habits as well as on both nutritional and functional properties [27,28,29]. Therefore, we cannot conclude that the differences in our study seen between the two different growth habits are also not dependent on these conditions. Nevertheless, the growth habit seemed to influenced the fiber content, where the ascending cultivars, *Saigua*, have the highest content, up to 12%, compared to 7–8% for the decumbent cultivars, where the high fiber content could be related to ascending plants as seen in other studies [30]. 

Total carbohydrate contents (61–68%) were lower than that of common cereals (60–75%) [31], mainly for ascending (*Saigua*) whole grains, which had slightly lower values (57%). Starch is the most abundant carbohydrate in pseudocereals [32]. 

Dehulling affects mineral content (Table 2 and Table 3, Figure 3A,B). Similar observations were previously reported in quinoa, where Ca and K contents decreased with dehulling. Nevertheless, in our study, the effect was extended to the content of macro-minerals, Ca, K, Na, P, and Mg, in decreasing order [33]. Our findings suggest that these minerals are found mainly in the pericarp and the internal layers of the grain, as suggested by other authors when explaining a similar observation in quinoa [33,34]. 

Micro-minerals such as Fe, Zn, and Cu are comparably high in canihua to what is found in quinoa [35]. It is generally observed that the mineral content is favored in whole grains, where the fibers in the pericarp consist partly of anionic material that interacts strongly with the minerals. Phytic acid may also be associated with iron and other polyvalent cations [17]. As previously found for amaranth, there were no negative relationships where Fe and Na showed a negative correlation [21]. The highest Fe content is for the *Saigua* (ascending) cultivars, about 20–30 mg/100 g DM, which can make it a more suitable food for handling iron deficiency than common cereals [36], a disease that the FAO describes as problematic, especially in pregnant women who are still malnourished and overweight [37]. However, it is also necessary to pay attention to antinutrient factors as well [17]. Dehulling did not affect the Zn content like that found for quinoa [33]. In addition, the Zn content is similar between different cultivars and growth habits.

The mineral content was not related to the coloration of the grains, as other authors have found for amaranth [38]. However, it was possible to appreciate that the colorations are different in each case, and some studies of canihua and corn indicate that the intense coloration of the grains can be directly related to the content of secondary metabolites, i.e., phenolic compounds, carotenoids, and anthocyanins, with antioxidant functions [18,39]. Therefore, it would be interesting to expand the studies towards these analyses.

In general, fatty acids (FA) were not affected by dehulling (Figure 3C), so they would be present mainly in the embryo and endosperm [40]. Other authors found that the FA content is affected by the origin of the grains [41], but in the present study, the origin did not differentiate between the grains and the growth habit (Figure 3C).

Canihua oil is a pretty unsaturated oil, and it has adequate proportions in terms of linoleic (ω6) and linolenic (ω3) acids, an ω6/ω3 ratio of about 10 [42]. Whole ascending canihua oil had a ω6/ω3 ratio of 10/1 (Table 4). The others were slightly above the range (11/1–12/1). It can be noted that canihua oil has an unusual content of tricosanoic acid (C23). Previously it has been observed in amaranth [14].

The breeding of canihua is at a stage of yield improvement under its native conditions, resulting in a fairly limited geographical distribution. Nevertheless, it stands out for its good nutritional qualities. The dehulling process may affect this nutritional value. Certain cultivars characterized by different growth habits also have different nutritional content. Moreover, the different places of origin (3) have not been considered since the cultivars have different origins.

## 5. Conclusions

The canihua cultivars presented higher protein, crude fiber, and mineral contents for the cultivars with ascending growth habit as compared to the decumbent ones. The dehulling process affected the mineral composition of the macro-minerals and the Fe and Mn contents of the micro-minerals. Furthermore, no negative correlations were found between the mineral components. The fatty acid profile revealed that the lipid content was mainly unsaturated with a ratio ω6/ω3 between 10 and 12, slightly affected by growth habit, with an unusually high tricosanoic acid content.

## Figures and Tables

**Table 1:** Characteristics and location of canihua (*Chenopodium pallidicaule)* cultivars used in the study ^†^.

Cultivar	Origin (Locality, Province, Department)	Cultivation Altitude	Growth Habit	Growth Cycle	Yield
		[masl]		[days]	[T/ha]
*Lasta Rosada*	Caquiaviri, Pacajes, La Paz	3900	Decumbent/*lasta*	160	0.50
*Illimani*	Khipakhipani, Viacha, Ingavi, La Paz	3857	Decumbent/*lasta*	160	0.80
*Kullaca*	Patacamaya, Aroma, La Paz	3700	Decumbent/*lasta*	150	0.70
*Cañawiri*	Khipakhipani, Viacha, Ingavi La Paz	3857	Decumbent/*lasta*	122	0.90
*Saigua L24*	Khipakhipani, Viacha, Ingavi, La Paz	3857	Ascending/*saigua*	135	0.90
*Saigua L25*	Khipakhipani, Viacha, Ingavi, La Paz	3857	Ascending/*saigua*	135	0.90

^†^ These data were provided by PROINPA, Cochabamba, Bolivia.

**Table 2:** Proximate composition of canihua (*Chenopodium pallidicaule*) cultivars. All values [g/100 g] are presented in dry weight (dw), as mean ± standard deviation (*n* = 3). For each compound, different superscript lowercase letters in rows indicate significant differences between canihua samples (*p* < 0.05), and different uppercase letters indicate a significant difference between whole or dehulled grains.

	Whole Canihua						Dehulled Canihua					
	*Lasta Rosada*	*Illimani*	*Kullaca*	*Cañawiri*	*Saigua L24*	*Saigua L25*	*Lasta Rosada*	*Illimani*	*Kullaca*	*Cañawiri*	*Saigua L24*	*Saigua L25*
Proximate [g/100 g]												
Protein	15.86 ± 0.50 ^bB^	16.53 ± 0.60 ^bcBC^	17.83 ± 0.82 ^cC^	13.92 ± 0.15 ^aA^	16.94 ± 0.09 ^bcBC^	19.56 ± 0.76 ^dD^	15.65 ± 0.23 ^bB^	17.17 ± 0.45 ^bcC^	17.79 ± 0.62 ^cC^	12.52 ± 0.18 ^aA^	16.89 ± 0.67 ^bcBC^	18.12 ± 0.18 ^cdC^
Fat	9.67 ± 0.03 ^cdBC^	9.40 ± 0.14 ^abcAB^	9.75 ± 0.33 ^cdBC^	9.96 ± 0.26 ^cdeC^	9.10 ± 0.17 ^abA^	9.98 ± 0.09 ^deC^	10.32 ± 0.25 ^efC^	9.04 ± 0.10 ^aA^	9.66 ± 0.15 ^bcdB^	10.38 ± 0.24 ^efC^	9.73 ± 0.07 ^cdB^	10.58 ± 0.23 ^fC^
Ash	3.54 ± 0.08 ^cA^	4.08 ± 0.09 ^dB^	4.14 ± 0.04 ^dB^	4.03 ± 0.01 ^dB^	4.75 ± 0.05 ^eC^	5.12 ± 0.13 ^fD^	2.42 ± 0.07 ^aA^	2.55 ± 0.03 ^aA^	2.48 ± 0.07 ^aA^	2.52 ± 0.04 ^aA^	2.97 ± 0.12 ^bB^	2.97 ± 0.05 ^bB^
Crude fiber	7.19 ± 0.21 ^abcA^	8.13 ± 0.13 ^deBC^	7.70 ± 0.36 ^cdeABC^	7.47 ± 0.30 ^bcdAB^	12.5 ± 0.30 ^gD^	8.40 ± 0.14 ^eC^	7.11 ± 0.33 ^abcAB^	6.73 ± 0.22 ^abAB^	6.59 ± 0.21 ^aA^	6.98 ± 0.11 ^abcAB^	11.0 ± 0.45 ^fC^	7.46 ± 0.26 ^bcdB^
Carbohydrates	63.75 ± 0.31 ^eC^	61.86 ± 0.58 ^cdB^	60.57 ± 0.45 ^bcB^	64.62 ± 0.63 ^eC^	56.76 ± 0.09 ^aA^	56.94 ± 0.68 ^aA^	64.49 ± 0.62 ^eC^	64.50 ± 0.32 ^eC^	63.48 ± 0.60 ^deC^	67.59 ± 0.38 ^fD^	59.55 ± 0.80 ^bA^	61.29 ± 0.84 ^cB^
Starch	51.38 ± 0.59 ^bcBC^	50.95 ± 1.1 ^bcBC^	52.15 ± 0.58 ^cdC^	51.99 ± 0.57 ^bcdC^	44.39 ± 0.58 ^aA^	49.15 ± 1.2 ^bB^	54.51 ± 0.57 ^deCD^	52.89 ± 0.57 ^cdBC^	54.63 ± 1.1 ^deCD^	56.02 ± 1.2 ^efD^	49.16 ± 1.7 ^bA^	50.35 ± 1.1 ^bcAB^

**Table 3:** Mineral composition of canihua (*Chenopodium pallidicaule*) cultivars. All values [mg/100 g] are presented in dry weight (dw), as mean ± standard deviation (*n* = 3). For each compound, different superscript lowercase letters in rows indicate significant differences between canihua samples (*p <* 0.05), and different uppercase letters indicate a significant difference between whole or dehulled grains (*p* < 0.05).

	Whole Canihua						Dehulled Canihua					
	*Lasta Rosada*	*Illimani*	*Kullaca*	*Cañawiri*	*Saigua L24*	*Saigua L25*	*Lasta Rosada*	*Illimani*	*Kullaca*	*Cañawiri*	*Saigua L24*	*Saigua L25*
Macro-minerals [mg/100 g]												
P	407.25 ± 6.6 ^fC^	375.14 ± 5.2 ^dA^	399.35 ± 6.0 ^eBC^	402.73 ± 1.6 ^fC^	390.01 ± 2.1 ^eB^	453.87 ± 1.4 ^gD^	273.42 ± 1.0 ^aA^	304.14 ± 1.5 ^bB^	278.03 ± 4.1 ^aA^	309.44 ± 6.2 ^bcBC^	300.08 ± 0.7 ^bB^	317.96 ± 1.6 ^cC^
Na	16.63 ± 0.13 ^gD^	12.89 ± 0.51 ^eB^	15.38 ± 0.26 ^fC^	16.78 ± 0.69 ^gD^	7.86 ± 0.08 ^bcA^	7.17 ± 0.21 ^abA^	9.10 ± 0.42 ^dCD^	6.70 ± 0.30 ^aA^	12.45 ± 0.36 ^eE^	8.73 ± 0.39 ^cdC^	6.80 ± 0.31 ^abA^	7.11 ± 0.26 ^abAB^
K	1099.54 ± 1.7 ^fA^	1327.50 ± 20 ^hC^	1222.83± 14 ^gB^	1131.92 ± 28 ^fA^	1293.63 ± 11 ^gC^	1402.57 ± 4.7 ^hD^	645.17 ± 7.3 ^bB^	789.46 ± 3.3 ^dD^	742.97 ±12 ^cC^	594.17 ± 3.5 ^aA^	840.59 ± 22 ^eE^	795.39 ± 6.0 ^dD^
Ca	71.17 ± 3.49 ^dA^	86.83 ± 2.8 ^fB^	74.59 ± 2.42 ^deA^	78.74 ± 3.00 ^eA^	97.45 ± 2.35 ^gC^	102.13 ± 2.36 ^gC^	40.07 ± 1.45 ^aA^	46.15 ± 0.93 ^abB^	51.44 ± 1.11 ^bC^	49.99 ± 0.61 ^bBC^	40.24 ± 0.38 ^aA^	63.26 ± 2.68 ^cD^
Mg	262.19 ± 3.9 ^fgAB^	247.77 ± 2.5 ^efA^	273.72 ± 3.1 ^gB^	262.76 ± 13 ^fgAB^	250.19 ± 4.1 ^fA^	262.37 ±1.4 ^fgAB^	201.81 ± 2.1 ^bB^	174.10 ± 3.2 ^aA^	221.67 ± 7.2 ^cdCD^	215.04 ± 1.7 ^bcC^	232.75 ± 4.6 ^deDE^	234.01 ± 5.3 ^deE^
Micro-minerals [mg/100 g]												
Zn	3.44 ± 0.16 ^bcdAB^	3.72 ± 0.15 ^defBC^	3.93 ± 0.04 ^fC^	3.38 ± 0.07 ^bcA^	3.21 ± 0.03 ^abA^	3.74 ± 0.11 ^efC^	3.53 ± 0.12 ^cdeB^	3.88 ± 0.09 ^fC^	3.91 ± 0.04 ^fC^	3.52 ± 0.10 ^cdeB^	2.95 ± 0.11 ^aA^	3.54 ± 0.09 ^cdeB^
Mn	5.28 ± 0.12 ^bA^	5.19 ± 0.15 ^bA^	6.73 ± 0.06 ^dC^	5.25 ± 0.15 ^bA^	5.72 ± 0.14 ^cB^	4.98 ± 0.22 ^bA^	3.88 ± 0.08 ^aB^	3.51 ± 0.09 ^aA^	4.95 ± 0.12 ^bC^	3.53 ± 0.06 ^aA^	5.04 ± 0.21 ^bC^	3.82 ± 0.06 ^aAB^
Fe	13.49 ± 0.54 ^bA^	17.28 ± 0.52 ^cdB^	20.76 ± 0.42 ^efC^	19.78 ± 0.71 ^efC^	24.61 ± 0.41 ^gD^	27.86 ± 0.39 ^hE^	9.63 ± 0.46 ^aA^	13.34 ± 0.25 ^bB^	16.45 ± 0.33 ^cC^	17.70 ± 0.45 ^cdCD^	18.27 ± 0.44 ^deD^	22.00 ± 1.00 ^fE^
Cu	1.02 ± 0.04 ^dC^	1.30 ± 0.05 ^gD^	1.54 ± 0.04 ^hE^	1.08 ± 0.02 ^deC^	0.80 ± 0.03 ^bB^	0.55 ± 0.01 ^aA^	0.91 ± 0.03 ^cC^	1.22 ± 0.05 ^fgD^	1.16 ± 0.04 ^efD^	0.91 ± 0.01 ^cBC^	0.83 ± 0.02 ^bcB^	0.50 ± 0.01 ^aA^
Color												
L*	48.09 ± 0.01 ^jD^	49.92 ± 0.06 ^lF^	47.57 ± 0.06 ^iC^	48.52 ± 0.02 ^kE^	44.15 ± 0.01 ^gA^	45.90 ± 0.01 ^hB^	29.09 ± 0.63 ^cC^	30.13 ± 0.01 ^dD^	27.82 ± 0.02 ^bB^	33.18 ± 0.04 ^eE^	26.78 ± 0.02 ^aA^	35.54 ± 0.01 ^fF^
a*	1.80 ± 0.01 ^cB^	1.77 ± 0.02 ^cB^	1.77 ± 0.02 ^cB^	3.02 ± 0.01 ^dC^	0.55 ± 0.01 ^bA^	3.42 ± 0.03 ^eD^	5.32 ± 0.01 ^fB^	6.02 ± 0.01 ^gC^	7.16 ± 0.02 ^iE^	7.76 ± 0.04 ^jF^	0.07 ± 0.00 ^aA^	7.03 ± 0.02 ^hD^
b*	10.99 ± 0.04 ^eA^	11.66 ± 0.05 ^aC^	11.66 ± 0.04 ^fC^	15.19 ± 0.02 ^iE^	11.38 ± 0.03 ^fB^	12.69 ± 0.01 ^gD^	7.52 ± 0.02 ^cC^	7.36 ± 0.01 ^bB^	8.53 ± 0.02 ^dD^	14.46 ± 0.01 ^dF^	4.35 ± 0.02 ^aA^	13.17 ± 0.01 ^hE^

A broken vertical line between whole or dehulled samples differentiates decumbent habit from ascending growth habit.

**Table 4:** Fatty acid composition of canihua (*Chenopodium pallidicaule*) cultivars. All values [%] are presented in dry weight (dw), as mean ± standard deviation (*n* = 3). For each compound, different superscript lowercase letters in rows indicate significant differences between canihua samples (*p < 0.05*), and different uppercase letters indicate a significant difference between whole or dehulled grains (*p < 0.05*).

	Whole Canihua						Dehulled Canihua					
	*Lasta Rosada*	*Illimani*	*Kullaca*	*Cañawiri*	*Saigua L24*	*Saigua L25*	*Lasta Rosada*	*Illimani*	*Kullaca*	*Cañawiri*	*Saigua L24*	*Saigua L25*
Palmitic C16:0	14.63 ± 0.40 ^aA^	14.25 ± 0.48 ^aA^	13.37 ± 0.17 ^aA^	14.02 ± 1.3 ^aA^	13.28 ± 0.75 ^aA^	13.37 ± 1.0 ^aA^	13.40 ± 0.29 ^aA^	14.02 ± 0.03 ^aA^	13.71 ± 0.81 ^aA^	13.38 ± 0.39 ^aA^	13.77 ± 0.79 ^aA^	13.48 ± 0.20 ^aA^
Stearic C18:0	1.24 ± 0.22 ^abA^	1.18 ± 0.05 ^abA^	1.29 ± 0.13 ^abA^	ND	1.05 ± 0.0 ^aA^	1.06 ± 0.02 ^aA^	1.43 ± 00 ^bA^	1.44 ± 0.01 ^bA^	1.17 ± 0.22 ^abA^	1.15 ± 0.14 ^bcA^	1.11 ± 0.14 ^abA^	1.12 ± 0.14 ^abA^
Oleic C18:1	24.77 ± 0.66 ^bcBC^	25.26 ± 0.30 ^bcdCD^	24.43 ± 0.06 ^bB^	25.75 ± 0.52 ^cdD^	22.65 ± 0.16 ^aA^	22.28 ± 0.23 ^aA^	25.57 ± 0.11 ^cdA^	25.80 ± 0.74 ^dA^	24.88 ± 0.45 ^bcdA^	24.79 ± 0.18 ^bcA^	21.76 ± 0.30 ^aB^	22.23 ± 0.30 ^aB^
Linoleic C18:2	51.97± 0.64 ^aA^	52.26 ± 0.67 ^aA^	53.18 ± 0.69 ^abAB^	53.66 ± 0.51 ^abcABC^	54.76 ± 0.64 ^bcdBC^	55.16 ± 0.65 ^cdC^	52.71 ± 0.67 ^aB^	52.10 ± 0.60 ^aB^	53.18 ± 1.0 ^abB^	52.69 ± 0.24 ^aB^	55.68 ± 0.39 ^dA^	55.84 ± 0.35 ^dA^
Linolenic C18:3	4.41 ± 0.12 ^aA^	4.54 ± 0.17 ^aA^	4.73 ± 0.12 ^aA^	4.77 ± 0.46 ^aA^	5.46 ± 0.16 ^bB^	4.87 ± 0.25 ^abAB^	4.34 ± 0.30 ^aA^	4.42 ± 0.23 ^aA^	4.86 ± 0.08 ^abA^	4.96 ± 0.09 ^abA^	4.76 ± 0.28 ^aB^	4.63 ± 0.08 ^aB^
Arachidic C20:0	0.56 ± 0.11 ^bAB^	0.67 ± 0.08 ^bAB^	0.62 ± 0.08 ^bAB^	ND	0.51 ± 0.13 ^bA^	0.45 ± 0.03 ^bB^	0.66 ± 0.02 ^bA^	0.58 ± 0.11 ^bA^	0.69 ± 0.06 ^bA^	0.52 ± 0.04 ^bA^	0.54 ± 0.07 ^bA^	0.31 ± 0.02 ^abA^
Behenic C22:0	0.47 ± 0.01 ^bA^	0.51 ± 0.05 ^aA^	0.50 ± 0.04 ^aA^	ND	0.49 ± 0.09 ^aA^	0.51 ± 0.02 ^aA^	0.45 ± 0.04 ^aA^	0.46 ± 0.02 ^aA^	0.45 ± 0.03 ^aA^	0.40 ± 0.02 ^aA^	0.41 ± 0.02 ^aA^	0.48 ± 0.05 ^aA^
Erucic C22:1	0.37 ± 0.03 ^abABC^	0.28 ± 0.03 ^aA^	0.32 ± 0.01 ^aAB^	0.47 ± 0.01 ^bcBC^	0.44 ± 0.14 ^bcC^	0.33 ± 0.03 ^bAB^	0.34 ± 0.01 ^abBC^	0.28 ± 0.01 ^aA^	0.32 ± 0.01 ^aAB^	0.51 ± 0.01 ^cD^	0.36 ± 0.01 ^abBC^	0.37 ± 0.01 ^abC^
Tricosanoic C23:0	1.43 ± 0.04 ^bA^	0.77 ± 0.13 ^aA^	1.43 ± 0.02 ^bA^	1.33 ± 0.02 ^abA^	1.25 ± 0.06 ^abA^	1.49 ± 0.04 ^bA^	1.30 ± 0.04 ^abB^	0.51 ± 0.07 ^aB^	1.45 ± 0.11 ^bA^	1.43 ± 0.09 ^bB^	1.39 ± 0.03 ^bB^	1.54 ± 0.08 ^bB^
Lignoceric C24:0	0.22 ± 0.02 ^bA^	ND	0.25 ± 0.01 ^bA^	ND	ND	0.19 ± 0.01 ^aA^	ND	0.18 ± 0.01 ^a^	ND	0.15 ± 0.01 ^a^	ND	ND
PUFA	56.38 ± 0.53 ^aA^	56.79 ± 0.47 ^abAB^	57.91 ± 0.58 ^abAB^	58.42 ± 0.82 ^bcBC^	60.23 ± 0.52 ^cdD^	60.03 ± 0.87 ^cdCD^	57.05 ± 0.74 ^abA^	56.52 ± 0.46 ^bA^	58.03 ± 1.0 ^abA^	57.65 ± 0.23 ^bA^	60.45 ± 0.58 ^dB^	60.48 ± 0.35 ^dB^
UFA	81.15 ± 0.26 ^aA^	82.05 ± 0.26 ^aA^	82.34 ± 0.52 ^aA^	84.17 ± 1.3 ^aA^	82.88 ± 0.67 ^aA^	82.30 ± 1.0 ^aA^	82.62 ± 0.72 ^aA^	82.32 ± 1.0 ^aA^	82.92 ± 0.97 ^aA^	82.45 ± 0.40 ^aA^	82.21 ± 0.58 ^aA^	82.71 ± 0.40 ^aA^
SFA	18.48 ± 0.70 ^bB^	17.61 ± 0.24 ^bB^	17.48 ± 0.46 ^abAB^	15.36 ± 1.3 ^aA^	16.59 ± 0.54 ^abAB^	17.36 ± 1.0 ^abAB^	17.03 ± 0.70 ^abA^	18.06 ± 0.36 ^bA^	16.76 ± 0.96 ^abA^	17.04 ± 0.41 ^abA^	17.19 ± 0.87 ^abA^	16.93 ± 0.41 ^abA^
ɷ6/ɷ3	11.78 ± 0.45 ^bB^	11.53 ± 0.65 ^abAB^	11.24 ± 0.43 ^abAB^	11.32 ± 1.1 ^abAB^	10.03 ± 0.40 ^aA^	11.35 ± 0.45 ^abAB^	12.17 ± 0.86 ^bB^	11.80 ± 0.74 ^bAB^	10.95 ± 0.18 ^abAB^	10.62 ± 0.22 ^abA^	11.72 ± 0.22 ^abAB^	12.05 ± 0.22 ^bAB^

ND: not detected by the method used. A broken vertical line between whole or dehulled samples differs decumbent habit from ascending growth habit.

## Data Availability

Not applicable.
